# Genome-wide variance quantitative trait locus analysis suggests small interaction effects in blood pressure traits

**DOI:** 10.1038/s41598-022-16908-7

**Published:** 2022-07-25

**Authors:** Gang Shi

**Affiliations:** School of Telecommunications Engineering, Xidian University, 2 South Taibai Road, Xi’an, 710071 Shaanxi China

**Keywords:** Genetic association study, Quantitative trait

## Abstract

Genome-wide variance quantitative trait loci (vQTL) analysis complements genome-wide association study (GWAS) and has the potential to identify novel variants associated with the trait, explain additional trait variance and lead to the identification of factors that modulate the genetic effects. I conducted genome-wide analysis of the UK Biobank data and identified 27 vQTLs associated with systolic blood pressure (SBP), diastolic blood pressure (DBP) and pulse pressure (PP). The top single-nucleotide polymorphisms (SNPs) are enriched for expression QTLs (eQTLs) or splicing QTLs (sQTLs) annotated by GTEx, suggesting their regulatory roles in mediating the associations with blood pressure (BP). Of the 27 vQTLs, 14 are known BP-associated QTLs discovered by GWASs. The heteroscedasticity effects of the 13 novel vQTLs are larger than their genetic main effects, which were not detected by existing GWASs. The total R-squared of the 27 top SNPs due to variance heteroscedasticity is 0.28%, compared with 0.50% owing to their main effects. The overall effect size of the variance heteroscedasticity is small in GWAS SNPs compared with their main effects. For the 411, 384 and 285 GWAS SNPs associated with SBP, DBP and PP, respectively, their heteroscedasticity effects were 0.52%, 0.43%, and 0.16%, and their main effects were 5.13%, 5.61%, and 3.75%, respectively. The number and effects of the vQTLs are small, which suggests that the effects of gene–environment and gene–gene interactions are small. The main effects of the SNPs remain the major source of genetic variance for BP, which would probably be true for other complex traits as well.

## Introduction

Variance quantitative trait locus (vQTL) refers to a locus that is associated with the difference in the variance in a quantitative trait^1–4^. Such variance heteroscedasticity may be induced by gene–environment interactions^2,5–8^, gene–gene interactions^4^, multiple linked functional variants at the locus^9,10^ or scale effects^11,12^. Since standard genome-wide association studies (GWASs) focus on testing differences in the means across genotypes, genetic variance attributable to variance heteroscedasticity is missed. Genome-wide vQTL analysis complements GWASs and has the potential to identify novel variants associated with a trait. In addition, the variance heteroscedasticity of a vQTL is genotype-dependent, therefore, could explain additional trait variance that is genetically related. Without the need to measure environmental factors, genome-wide vQTL analysis has been used to screen potential gene–environment interactions and search for factors that modulate the genetic effects^2,5,6,8^.

To date, the identified vQTLs are largely for obesity-related traits. Yang et al. found that the *FTO* gene locus was not only associated with the mean of body mass index (BMI), but also the variance of it^13^. In the analysis of BMI with UK biobank data, Young et al. identified 48 genome-wide significant loci that demonstrated smaller P values from the test including variance heteroscedasticity effect than from the test of additive effect in trait mean only^5^. In the analysis of 13 quantitative traits from the UK Biobank, Wang et al. discovered 75 significant vQTLs for 9 traits, 60 of which were for those related to obesity^6^. Their interaction analysis showed that the vQTLs were enriched with gene-environment interactions. In the analysis of the genetic risk score of BMI with 376 variants, Sulc et al. demonstrated that while the genetic risk score explained 5.2% of BMI variance, its interactions with environmental factors explained an additional 1.9%^7^. Marderstein et al. showed that the discovery and replication rates of gene-environment interactions for BMI were significantly higher when prioritizing variants in vQTLs compared to when accessing all genetic variants. They also demonstrated strong gene-environment interactions mediated the genetic contribution to body weight and diabetes risk.

A recent study of blood pressure (BP) shows that some portions of the BP variance could be attributed to gene–environment interactions^14^. In this work, I propose statistical methods for vQTL analysis at the biobank scale that are based on a linear mixed model and regressions. I conducted genome-wide vQTL analysis of BP data in the UK Biobank to search for novel single-nucleotide polymorphisms (SNPs) associated with BP and evaluated additional BP variance explained by their variance heteroscedasticity.

## Material and methods

### Mixed model analysis

A variety of heteroscedasticity tests have been suggested for finding vQTLs, which have been reviewed previously^11,15^. In this work, I employ a linear mixed model and test the variance heteroscedasticity of a vQTL using the maximum likelihood approach, similar to that in (Sulc et al., 2020)^7^. Nevertheless, it focuses on testing the variance heteroscedasticity due to the interactions between a polygenic score and unknown environmental factors. The method is statistically powerful when the quantitative trait approximately follows a normal distribution.

Suppose that a quantitative trait *y* is associated with a genetic factor *G* and *n* covariates *X*_*i*_, *i* = 1,…,*n*, as follows:

$$ y = \mathop \sum \limits_{i = 1}^{n} \beta_{i}^{{\text{C}}} X_{i} + \beta^{{\text{G}}} G + \mathop \sum \limits_{i = 1}^{m} \beta_{i}^{{\text{E}}} E_{i} + \mathop \sum \limits_{i = 1}^{m} \beta_{i}^{{\text{I}}} E_{i} G + \varepsilon, $$

where $\beta^{{\text{G}}}$ and $\beta_{i}^{{\text{C}}}$, *i* = 1,…,*n*, are the genetic and covariate effects, respectively, and $\varepsilon \sim {\text{N}}\left( {0,\sigma^{2} } \right)$ is the random error. Here, *G* is assumed to be additive, which could be the dosage or coded genotype of a SNP. *E*_*i*_, *i* = 1,…,*m* are *m* environmental factors that modulate the genetic effect, and $\beta_{i}^{{\text{E}}}$ and $\beta_{i}^{{\text{I}}}$ are the environmental and interaction effects, respectively. Without loss of generality, *E*_*i*_, *i* = 1,…,*m* may include other factors that modulate the genetic effect.

Letting $\gamma_{1} = \mathop \sum \limits_{i = 1}^{m} \beta_{i}^{{\text{E}}} E_{i}$ and $\gamma_{2} = \mathop \sum \limits_{i = 1}^{m} \beta_{i}^{{\text{I}}} E_{i}$, I have

$$ y = \mathop \sum \limits_{i = 1}^{n} \beta_{i}^{{\text{C}}} X_{i} + \beta^{{\text{G}}} G + \gamma_{1} + \gamma_{2} G + \varepsilon. $$

Assume that *E*_*i*_, *i* = 1,…,*m* are centralized and uncorrelated, conditional on the covariates and genetic factor, trait variance depends on the genetic factor in a quadratic manner

$$ {\text{var}}\left( y \right) = \tau_{1} + 2\tau_{2} G + \tau_{3} G^{2} + \sigma^{2}, $$

where

$$ \tau_{1} = {\text{var}}\left( {\gamma_{1} } \right) = \mathop \sum \limits_{i = 1}^{m} \left( {\beta_{i}^{{\text{E}}} } \right)^{2} {\text{var}}\left( {E_{i} } \right) $$

$$ \tau_{2} = {\text{cov}}\left( {\gamma_{1},\gamma_{2} } \right) = \mathop \sum \limits_{i = 1}^{m} \beta_{i}^{{\text{E}}} \beta_{i}^{{\text{I}}} {\text{var}}\left( {E_{i} } \right), $$

$$ \tau_{3} = {\text{var}}\left( {\gamma_{2} } \right) = \mathop \sum \limits_{i = 1}^{m} \left( {\beta_{i}^{{\text{I}}} } \right)^{2} {\text{var}}\left( {E_{i} } \right). $$

I further assume that $\gamma_{1}$ and $\gamma_{2}$ follow a bivariate normal distribution

$$ \left( {\begin{array}{*{20}c} {\gamma_{1} } \\ {\gamma_{2} } \\ \end{array} } \right)\sim {\text{N}}\left[ {\left( {\begin{array}{*{20}c} 0 \\ 0 \\ \end{array} } \right),\left( {\begin{array}{*{20}c} {\tau_{1} } & {\tau_{2} } \\ {\tau_{2} } & {\tau_{3} } \\ \end{array} } \right)} \right]. $$

Note that $\sigma^{2}$ and $\tau_{1}$ are not identifiable in the present model. I merge the error $\varepsilon$ with the random effect $\gamma_{1}$ as the new $\gamma_{1}$ and have the linear mixed model

1 $$ y = \mathop \sum \limits_{i = 1}^{n} \beta_{i}^{{\text{C}}} X_{i} + \beta^{{\text{G}}} G + \gamma_{1} + \gamma_{2} G, $$

whose mean and variance are

$$ {\text{E}}\left( y \right) = \mathop \sum \limits_{i = 1}^{n} \beta_{i}^{{\text{C}}} X_{i} + \beta^{{\text{G}}} \beta_{{\text{G}}}, $$

$$ {\text{var}}\left( y \right) = \tau_{1} + 2\tau_{2} G + \tau_{3} G^{2}. $$

In this case, $\tau_{1}$ is the variance of $\gamma_{1} + \varepsilon$ in the original model, and $\tau_{2}$ is the covariance between $\gamma_{2}$ and $\gamma_{1} + \varepsilon$.

I solve the linear mixed model (1) numerically by the maximum likelihood method based on its profiled likelihood function and Newton–Raphson algorithm^16^. To alleviate computational burden, one can also first regress the trait on the covariates and genetic factor and then use the residual $\hat{e}$ to solve the variance component model

2 $$ \hat{e} = \gamma_{1} + \gamma_{2} G. $$

To test the variance heteroscedasticity, the null hypothesis is H_0_: $\tau_{2} = \tau_{3} = 0$, and the alternative hypothesis is that at least one of them is nonzero. The likelihood ratio statistics follows a 0.5:0.5 mixture distribution of a chi-square with 1 degree of freedom (df) and a chi-square with 2 df^17^. All tests proposed in this paper were implemented in the program “heter”, which is available at https://github.com/eat1000/heter.

### Regression analysis

To solve the linear mixed model (1) or the variance component model (2), it is usually desirable for the starting values of the iterating parameters to be close to their estimates. I extend the Breusch–Pagan test for heteroscedasticity^18^ into a quadratic form, and the squared residual is regressed on the genetic factor as follows:

3 $$ \hat{e}^{2} = \tau_{1} + 2\tau_{2} G + \tau_{3} G^{2} + \varepsilon, $$

where $\varepsilon$ is the random error. The estimated $\hat{\tau }_{1}$, $\hat{\tau }_{2}$ and $\hat{\tau }_{3}$ by linear regression can be used as the starting values of the variance parameters for solving model (1) or (2). Alternatively, the heteroscedasticity test can also be performed by linear regression (3) by testing $\tau_{2}$ and $\tau_{3}$ jointly, and the likelihood ratio statistics follows a Chi-square distribution with 2 df.

Since the squared residual $\hat{e}^{2}$ is nonnegative, linear regression (3), which assumes normally distributed $\varepsilon$, could be suboptimal. An improved Chi-square regression analysis is formulated as follows:

4 $$ \hat{e}^{2} = \left( {\tau_{1} + 2\tau_{2} G + \tau_{3} G^{2} } \right)\chi_{1}^{2}, $$

where $\chi_{1}^{2}$ is a random variable following a Chi-square distribution with 1 df. I iteratively solve the Chi-square regression (4) by the maximum likelihood method with starting values estimated by linear regression (3). The test for variance heteroscedasticity is conducted based on testing $\tau_{2}$ and $\tau_{3}$ jointly using the likelihood ratio test, and the test statistics follows a Chi-square distribution with 2 df. It can be shown that the likelihood function of Chi-square regression (4) coincides with that of the variance component model (2) and that of the gamma regression with a shape parameter of 1/2 and a scale parameter modeled as $2\tau_{1} + 4\tau_{2} G + 2\tau_{3} G^{2}$.

Notably, $\tau_{1}$, $\tau_{2}$ and $\tau_{3}$ are unconstrained when solving regression models (3) and (4), and no distributions of $\gamma_{1}$ and $\gamma_{2}$ have to be assumed. Nevertheless, $\gamma_{1}$ and $\gamma_{2}$ are assumed to follow a bivariate normal distribution in the linear mixed model (1) and variance component model (2), and $$\left( {\begin{array}{*{20}c} {\tau_{1} } & {\tau_{2} } \\ {\tau_{2} } & {\tau_{3} } \\ \end{array} } \right)$$ are constrained to be positive semidefinite.

### UK Biobank data and analyses

The UK Biobank is a large propective study in the United Kingdom with more than 500,000 participants aged between 40 and 69 years at the time of recruitment. The study design was described previously^19^. Deep phenotyping, genomic, and health-related data have been collected and are available for research investigating a wide range of diseases caused by a combination of genes, lifestyles, and environmental factors^20^. The UK Biobank has obtained informed consent from all participants and has obtained Research Tissue Bank approval from its ethics committee. This research has been approved by the UK Biobank under application number 44080 and I have complied with all relevant ethical regulations in this work.

Genome-wide genotype data of 487,422 individuals were imputed with the Haplotype Reference Consortium (HRC) reference panel by the UK Biobank, resulting in 93,095,623 autosomal SNPs, short indels and large structural variants^20^. I conducted genome-wide vQTL analysis of BP data in the UK Biobank using imputed genotype data. I restricted the analysis to autosomal SNPs that have minor allele frequencies higher than 0.01 and information scores larger than 0.9, obtaining 9,117,915 SNPs that passed the filters. I excluded quality control outliers for heterozygosity or missingness and samples with sex discordance between the self-reported and genetically inferred sex according to the sample quality control files provided by the UK Biobank. I also excluded non-European samples, samples from pregnant women, one sample in each related pair up to second-degree relatives, and samples from participants who had withdrawn consent.

Systolic blood pressure (SBP) and diastolic blood pressure (DBP) in the genome-wide vQTL analysis were averaged over multiple measurements assessed at baseline, which were further adjusted for antihypertensive medication use by adding 15 and 10 mm Hg^21^ to SBP and DBP, respectively. Pulse pressure (PP) was computed as the difference between SBP and DBP and then logarithmically transformed. Covariates in the analysis included sex, age, age squared, BMI and the top 10 principal components (PCs). Samples with BP or BMI values that were 5 or more standard deviations outside the respective means and those with missing BP values or covariates were also excluded. The total sample sizes in the analyses were 396,077, 396,079 and 396,077 for SBP, DBP and PP, respectively.

### Simulation studies

I conducted simulation studies to examine the distributions of the four test statistics under the null hypothesis and their statistical power to detect a vQTL with different heteroscedasticity effects. Covariates and genotype data in the UK Biobank were used for the simulation studies.

I simulated an SBP trait under the null hypothesis as follows:

$$ {\text{SBP}} = 86.5 + 5.65{\text{sex}} + 0.00827{\text{age}}^{2} + 0.909{\text{BMI}} + 0.0647{\text{PC}}_{4} + 0.0349{\text{PC}}_{9} + \mathop \sum \limits_{i = 1}^{54} \beta_{i} {\text{SNP}}_{i} + \varepsilon $$

in which the intercept, covariate effects and standard deviation of the random error $\varepsilon$ were from the fitted model by using the SBP and covariates in the UK Biobank, the 54 SBP-associated SNPs and their effects were as reported in the literature^22^, and $\varepsilon$ was normally distributed with a zero mean and standard deviation of 18.44. Genotypes of the SNPs were called as those with the largest probabilities and then coded as being additive. For the 396,387 samples who passed quality controls and had non-missing covariates, their SBP values were simulated. Since the simulated SNP effects are constants across samples, there is no variance heteroscedasticity in this simulation. Then, I carried out four vQTL analyses on 10,000 SNPs randomly chosen from SNPs on 22 autosomes that passed the quality controls.

To evaluate the statistical power of the vQTL tests, I assumed that the effect of one SBP-associated SNP, rs880315, was random, following a normal distribution ${\text{N}}\left( { - 0.475,\sigma^{2} } \right)$ in the simulated samples. Here, the mean −0.475 is the average effect per reference allele as reported in the literature^22^, and $\sigma^{2}$ is the variance of the effect. The effects of covariates and the other 53 SNPs remained the same as previously described. I considered 5 levels of heteroscedasticity with $\sigma^{2}$ = 1, 2, 3, 4, and 5. For each level, I simulated the SBP of 396,387 samples with 1000 replicates. I conducted the four vQTL analyses on SNP rs880315. The results of P values smaller than the genome-wide significance threshold of 5 × 10^−8^ ^23^ were considered to be significant, and a portion of the significant results among the 1000 replicates was the empirical power.

## Results

### Simulation results

**Figure 1:**
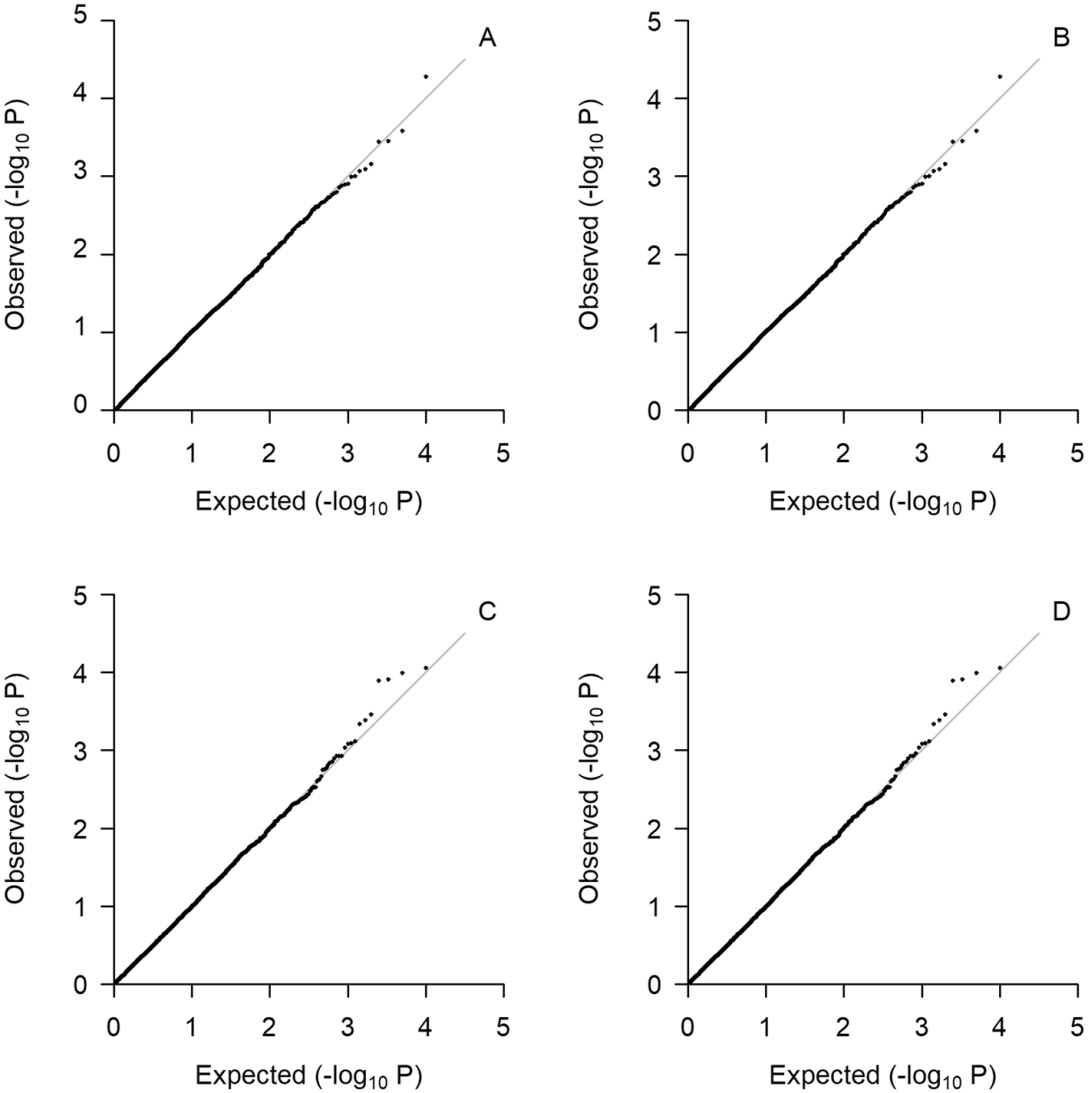
Quantile–quantile plots for the four test statistics under the null hypothesis. (**A**) Linear mixed model, (**B**) variance component model, (**C**) linear regression, (**D**) Chi-square regression.

Under the null hypothesis, quantile–quantile (QQ) plots of the four vQTL tests are presented in Fig. 1. The observed P values from testing the 10,000 SNPs are shown as the vertical coordinates on a negative log_10_ scale, and the horizontal coordinates are their expected values from a uniform distribution between 0 and 1. As can be seen, the empirical distributions and their expected distributions align well for the four tests. The QQ plots for the linear mixed model (1) and variance component model (2) are almost identical. I compared the likelihood ratio statistics of the two tests, the test statistics based on the linear mixed model (1) and the variance component model (2) are almost identical as well. This is probably because the maximum likelihood estimates of fixed effects in linear mixed models are robust to the misspecification of their covariance structure^24^. Even though the estimates of the fixed effects are updated in each iteration when solving the linear mixed model (1), the changes are minuscule. Without the loss of much precision, the fixed effects can be estimated and kept unchanged, and the parameters of the random effects are estimated iteratively, which is equivalent to solving the variance component model (2). Similarly, the QQ plots for linear regression (3) and Chi-square regression (4) are very close, which is possibly due to the robustness of the linear regression.

**Figure 2:**
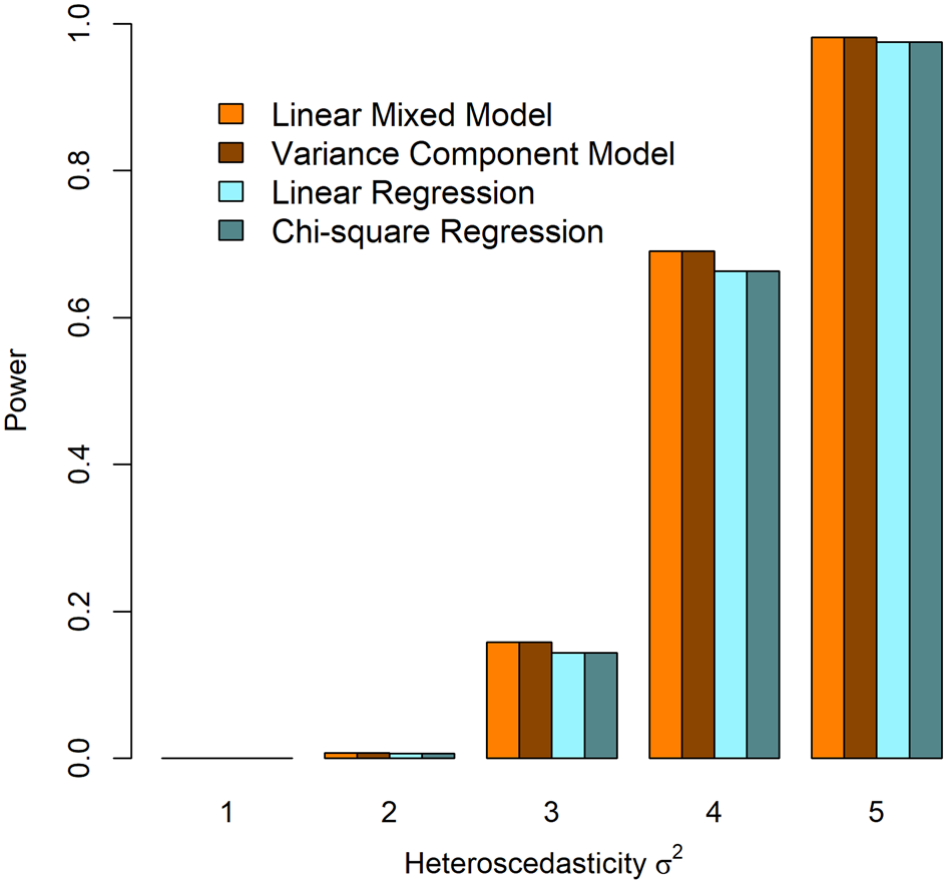
Statistical power of the four tests with different heteroscedasticity effects.

The statistical power of the four tests is displayed in Fig. 2. Apparently, the power of the four tests becomes large with the increase in the heteroscedasticity effect. The power based on the linear mixed model (1) and the variance component model (2) are the same, and the test statistics based on the linear mixed model (1) and the variance component model (2) are almost identical. The powers of the tests based on linear regression and Chi-square regression are also the same. Nevertheless, test statistics based on Chi-square regression (4) are slightly larger than those based on linear regression (3) for some replicates when the heteroscedasticity effect is large. Compared with the linear mixed model (1) and variance component model (2), linear regression (3) and Chi-square regression (4) are statistically less powerful. This is because the test statistics based on regressions have larger dfs. Since the variance component method has the same power as the linear mixed model and is computationally much less expensive, I used it as the primary analysis in the analysis of BP data in the UK Biobank.

### Genome-wide vQTL analysis of blood pressures

**Figure 3:**
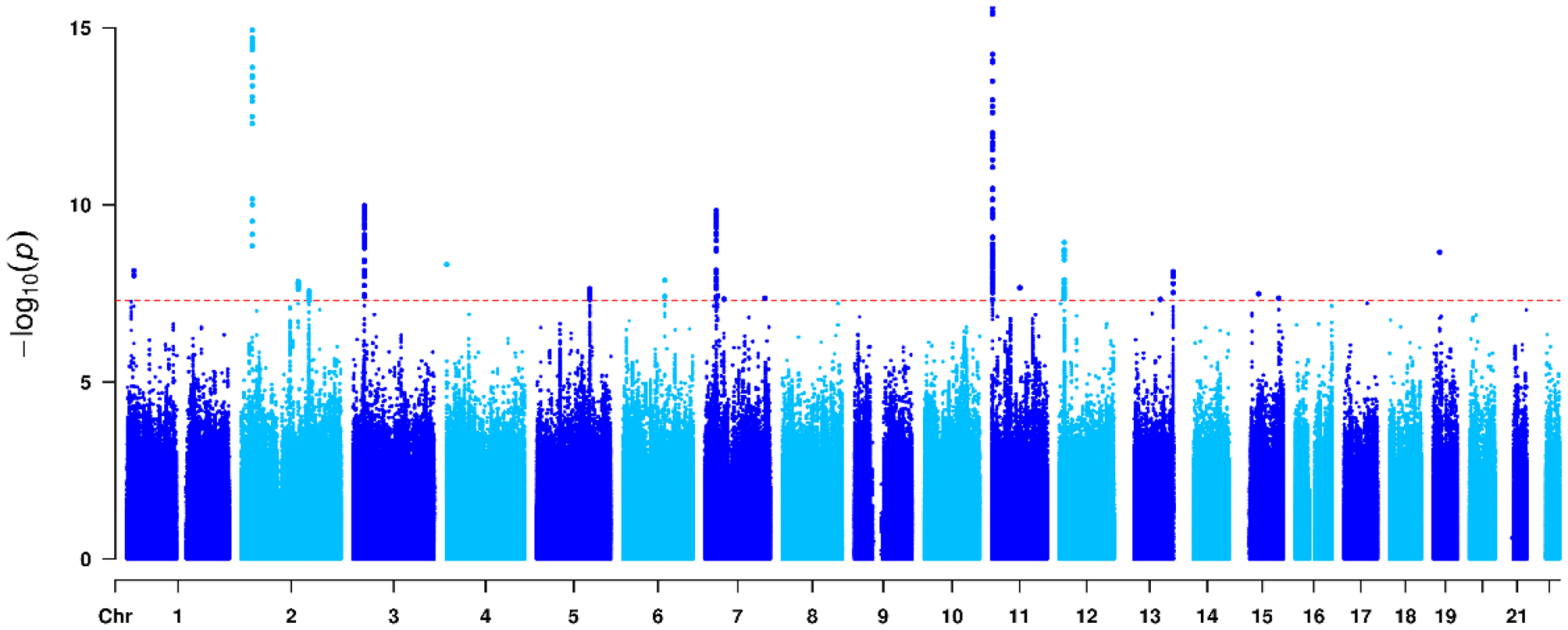
Manhattan plot visualizing genome-wide results from the vQTL analysis of SBP. Dots denote the SNPs in the genome-wide vQTL analysis, whose P values on a negative log_10_ scale are plotted against their physical positions. The dashed line represents the genome-wide significance level (P = 5 × 10^−8^).

**Figure 4:**
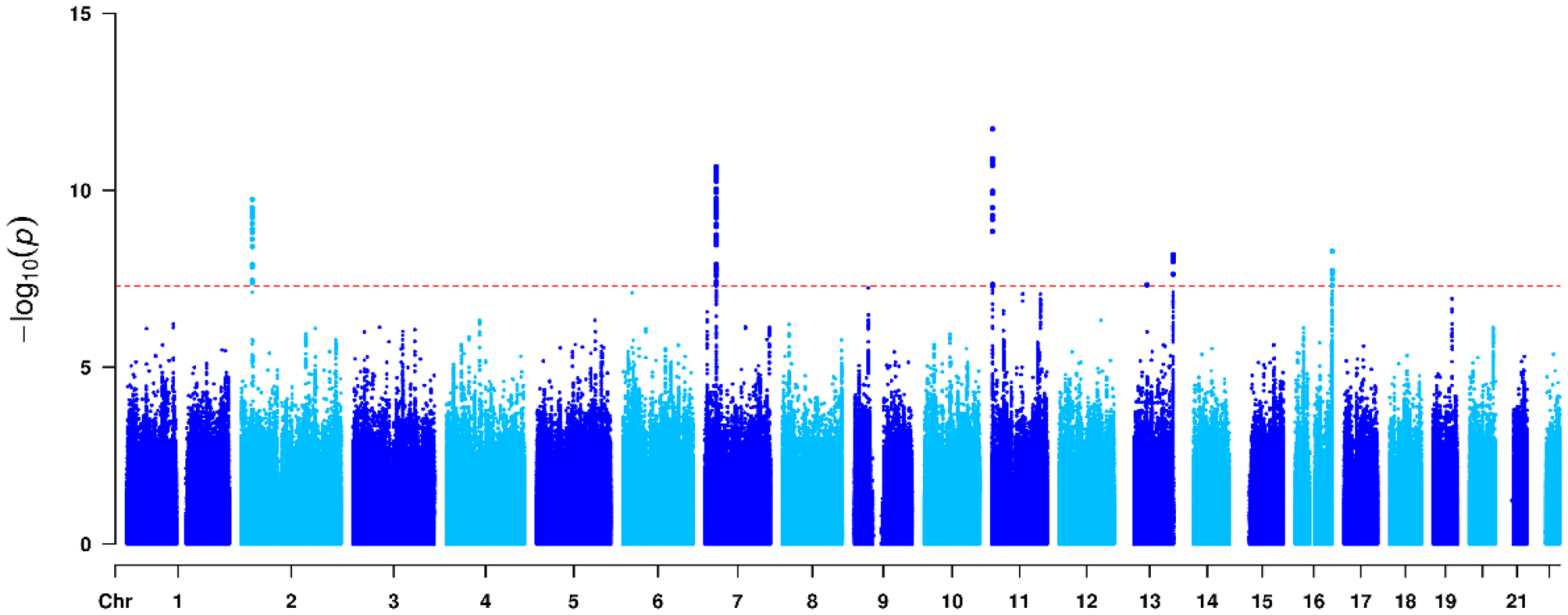
Manhattan plot visualizing genome-wide results from the vQTL analysis of DBP. Dots denote the SNPs in the genome-wide vQTL analysis, whose P values on a negative log_10_ scale are plotted against their physical positions. The dashed line represents the genome-wide significance level (P = 5 × 10^−8^).

**Figure 5:**
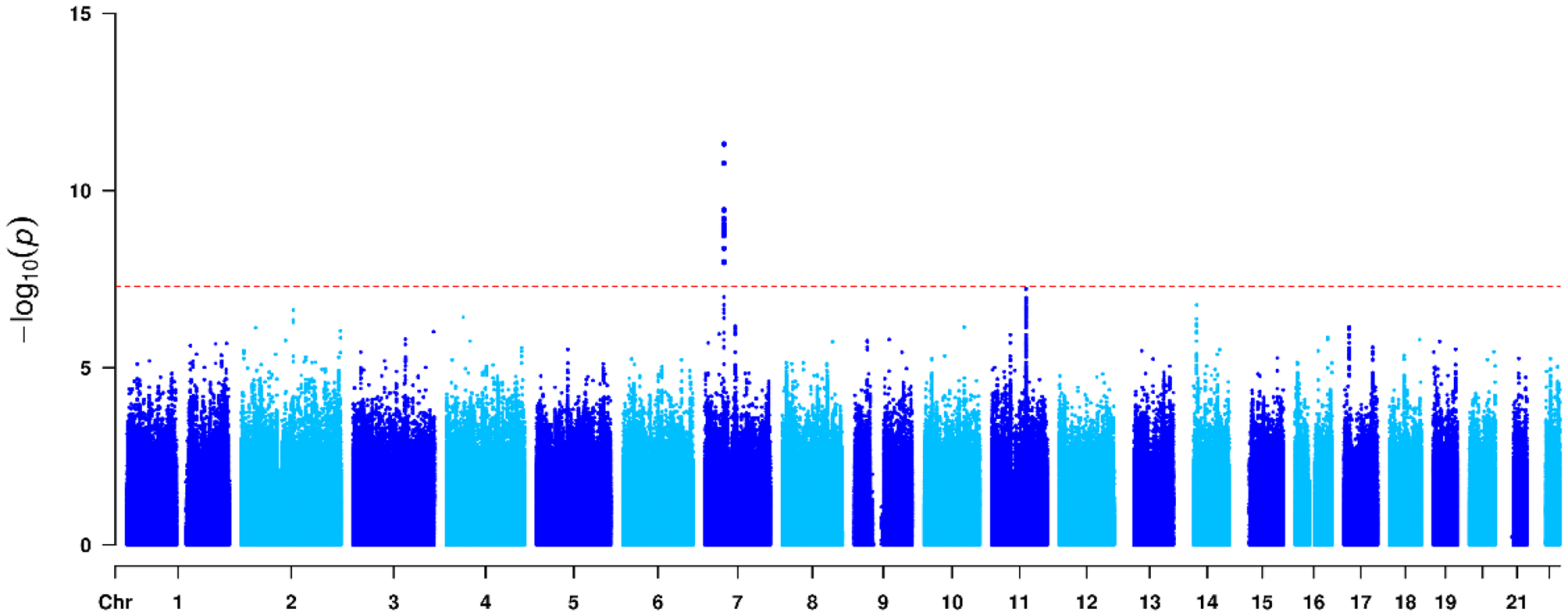
Manhattan plot visualizing genome-wide results from the vQTL analysis of PP. Dots denote the SNPs in the genome-wide vQTL analysis, whose P values on a negative log_10_ scale are plotted against their physical positions. The dashed line represents the genome-wide significance level (P = 5 × 10^−8^).

I conducted genome-wide vQTL analysis of BP data in the UK Biobank, and the results of the variance component model are displayed as Manhattan plots in Figs. 3, 4 and 5. I detected 20, 6 and 1 vQTLs that are associated with SBP, DBP and PP, respectively, at the genome-wide significance level (P < 5 × 10^−8^). The top SNPs that have the lowest P values at the loci are shown in Table 1. The results of the main effects of the SNPs were obtained from linear regression, as in standard GWAS. The *R*^2^ values of the heteroscedasticity effects were computed as the likelihood-ratio based pseudo-R-squared^25^.

**Table 1:** Genome-wide significant vQTLs associated with SBP, DBP, or PP.

Locus	Top SNP	Chr	Position	NCA/CA	CAF	Trait	Main effect			Heteroscedasticity		
							Effect	SE	P Value	*R*^2^	P Value	*R*^2^
***PADI2***	rs116515879	1	17,422,797	G/T	0.02	SBP	− 0.2868	0.1483	5.01 × 10^−2^	9.45 × 10^−6^	7.32 × 10^−9^	9.17 × 10^−5^
*CIB4-KCNK3*	rs1275984*^†^	2	26,911,509	A/C	0.62	SBP	− 0.6121	0.0427	1.33 × 10^−46^	5.19 × 10^−4^	1.15 × 10^−15^	1.71 × 10^−4^
						DBP	− 0.3205	0.024	1.03 × 10^−40^	4.51 × 10^−4^	1.80 × 10^−10^	1.10 × 10^−4^
***HNMT***	rs3100721^†‡^	2	138,755,054	T/C	0.35	SBP	0.0566	0.0433	1.91 × 10^−1^	4.31 × 10^−6^	1.47 × 10^−8^	8.82 × 10^−5^
*FIGN-GRB14*	rs7607074^§†^	2	164,898,256	T/A	0.51	SBP	− 0.4084	0.0417	1.10 × 10^−22^	2.43 × 10^−4^	2.75 × 10^−8^	8.50 × 10^−5^
*SLC4A7-EOMES*	rs2029827*^†‡^	3	27,551,275	A/G	0.4	SBP	− 0.4596	0.0424	2.40 × 10^−27^	2.96 × 10^−4^	1.06 × 10^−10^	1.13 × 10^−4^
***SPON2***	rs111822223^†‡^	4	1,179,966	A/G	0.07	SBP	0.217	0.0808	7.12 × 10^−3^	1.82 × 10^−5^	4.90 × 10^−9^	9.37 × 10^−5^
***MINAR2-CHSY3***	rs11955088^†^	5	129,206,827	A/G	0.57	SBP	0.0476	0.0419	2.56 × 10^−1^	3.26 × 10^−6^	2.36 × 10^−8^	8.58 × 10^−5^
***GRIK2***	rs144326314	6	101,863,870	G/GTCAA	0.11	SBP	− 0.1517	0.0701	2.92 × 10^−2^	1.18 × 10^−5^	1.36 × 10^−8^	8.86 × 10^−5^
*HOXA13-EVX1*	rs115525024^§^	7	27,236,559	T/G	0.93	DBP	0.5121	0.0447	2.46 × 10^−30^	3.31 × 10^−4^	2.14 × 10^−11^	1.21 × 10^−4^
	rs10262140^§†‡^	7	27,256,464	T/C	0.94	SBP	0.977	0.0862	9.34 × 10^−30^	3.24 × 10^−4^	1.45 × 10^−10^	1.11 × 10^−4^
***CRHR2***	rs41413147^†^	7	30,723,336	C/T	0.01	SBP	− 0.6249	0.19	9.93 × 10^−4^	2.73 × 10^−5^	3.75 × 10^−8^	8.35 × 10^−5^
*IGFBP3-TNS3*	rs11977526*^†‡^	7	46,008,110	G/A	0.4	SBP	− 0.3802	0.0423	2.54 × 10^−19^	2.04 × 10^−4^	4.64 × 10^−8^	8.24 × 10^−5^
						PP	− 0.0085	0.0005	6.33 × 10^−66^	7.42 × 10^−4^	4.86 × 10^−12^	1.29 × 10^−4^
***TPK1-CNTNAP2***	rs574733666	7	145,677,500	AT/A	0.09	SBP	0.1299	0.0742	7.42 × 10^−2^	7.74 × 10^−6^	4.37 × 10^−8^	8.27 × 10^−5^
*LSP1*	rs569550*^†‡^	11	1,887,068	T/G	0.39	SBP	0.6291	0.0427	3.49 × 10^−49^	5.48 × 10^−4^	4.29 × 10^−17^	1.87 × 10^−4^
						DBP	0.276	0.024	1.16 × 10^−30^	3.34 × 10^−4^	1.83 × 10^−12^	1.33 × 10^−4^
***PPP6R3***	rs547525853	11	68,243,714	C/T	0.01	SBP	0.3915	0.2036	5.14 × 10^−2^	9.33 × 10^−6^	2.17 × 10^−8^	8.62 × 10^−5^
*DUSP16*	rs12368847*^†‡^	12	12,682,123	G/A	0.27	SBP	− 0.2912	0.0469	5.25 × 10^−10^	9.74 × 10^−5^	1.16 × 10^−9^	1.01 × 10^−4^
***DLEU1-DLEU7***	rs77827164	13	51,174,271	G/A	0.02	DBP	− 0.0409	0.0869	6.38 × 10^−1^	5.60 × 10^−7^	4.73 × 10^−8^	8.23 × 10^−5^
***LINC00564-SLITRK1***	rs141943794	13	83,713,849	A/T	0.02	SBP	0.0189	0.1596	9.06 × 10^−1^	3.56 × 10^−8^	4.69 × 10^−8^	8.24 × 10^−5^
*LINC00552-TMEM255B*	rs376861852^§^	13	114,454,081	G/T	0.36	SBP	0.0799	0.0432	6.03 × 10^−2^	8.64 × 10^−6^	7.79 × 10^−9^	9.14 × 10^−5^
						DBP	0.1385	0.0243	1.13 × 10^−8^	8.23 × 10^−5^	6.63 × 10^−9^	9.22 × 10^−5^
***TYRO3-MGA***	rs182202119	15	41,918,628	A/T	0.01	SBP	0.3606	0.1964	6.22 × 10^−2^	8.51 × 10^−6^	3.29 × 10^−8^	8.41 × 10^−5^
*FURIN*	rs8032315^§†‡^	15	91,418,297	T/A	0.32	SBP	0.7136	0.0444	4.58 × 10^−58^	6.51 × 10^−4^	4.35 × 10^−8^	8.27 × 10^−5^
***SPIRE2***	rs34169212	16	89,912,736	C/CT	0.55	DBP	0.1212	0.0237	3.00 × 10^−7^	6.63 × 10^−5^	5.31 × 10^−9^	9.33 × 10^−5^
***WIZ***	rs113267381	19	15,550,532	G/A	0.02	SBP	− 0.1549	0.175	3.76 × 10^−1^	1.98 × 10^−6^	2.18 × 10^−9^	9.78 × 10^−5^

Novel loci are highlighted in bold. Positions are given in GRCh37 coordinates. SNP effects are in mm Hg per copy of the coded allele. *The SNP is, or in linkage disequilibrium (LD *R*^2^ ≥ 0.8) with, a BP-associated SNP in the GWAS catalog. ^†^The SNP is an eQTL SNP in GTEx. ^‡^The SNP is an sQTL SNP in GTEx. §The SNP is in intermediate LD (0.1 ≤ LD *R*^2^ < 0.8) with a BP-associated SNP in the GWAS catalog. Chr, chromosome; NCA, noncoded allele; CA, coded allele; CAF, coded allele frequency, SE, standard error.

The 27 significant vQTLs included the 23 top SNPs from 22 genomic loci, of which 10 SNPs from 9 loci showed significant SNP main effects (P < 5 × 10^−8^). rs1275984, rs11977526 and rs569550 are known BP-associated SNPs that are included in the GWAS catalog^26^. rs2029827 and rs12368847 are in linkage disequilibrium (LD *R*^2^ ≥ 0.8) with rs820430 and rs11609905, respectively, which are associated with BPs. rs7607074, rs115525024, rs10262140, rs376861852 and rs8032315 are in intermediate LD (0.1 ≤ LD *R*^2^ < 0.8) with the BP-associated SNPs rs16849211, rs7812039, rs7812039, rs3934939, and rs17514846, respectively, and their LD *R*^2^ ranges from 0.19 to 0.45.

The SNP main effects of the 27 vQTLs explain 0.50% of the BP variances collectively, and their heteroscedasticity accounts for an additional 0.28%, which is approximately half the variance attributed to the main effects. Because the vQTLs were discovered by the heteroscedasticity test, the relative contribution of the variance heteroscedasticity would be larger than that of all BP QTLs. I queried the GWAS catalog^26^ for SNPs with genome-wide significance that were discovered among samples of European ancestry and were replicated. There were 440, 395 and 305 SNPs reported to be associated with SBP (EFO_0006335), DBP (EFO_0006336) and PP (EFO_0005763), respectively, of which 411, 384 and 285 SNPs were present in the analysis. The sums of the SNP main effects were 5.13%, 5.61%, and 3.75%, and the heteroscedasticity effects were 0.52%, 0.43%, and 0.16% for SBP, DBP and PP, respectively. The contributions of the heteroscedasticity relative to the genetic main effects are approximately 0.1 for SBP and DBP and 0.04 for PP. Similarly, the relative contributions of the heteroscedasticity would be underestimated compared with that of all BP SNPs since the GWAS SNPs were largely detected by their genetic main effects. PP has the smallest relative contribution from the heteroscedasticity effects, possibly because PP is a derived trait and its random variation is larger than that in SBP and DBP.

Out of the 27 significant vQTLs, 13 are novel and are highlighted in Table 1. The P values of their genetic main effects ranged from 3.0 × 10^−7^ to 0.91 in this study and were not previously reported to be associated with BPs. The sizes of their SNP main effects, in terms of explained BP variance, are smaller than those of their heteroscedasticity effects. The total *R*^2^ values of the SNP main effects and heteroscedasticity effects are 0.017% and 0.11%, respectively. Clearly, it would be difficult to identify this type of locus in standard GWASs that focus on testing SNP main effects only.

The 27 vQTLs are enriched for genetic loci that affect the expression (eQTL) or splicing (sQTL) of protein-coding genes. Querying the GTEx data version 8^27^, 12 of the 23 top SNPs are eQTLs, and 8 top SNPs are both eQTLs and sQTLs. All of the eQTLs and sQTLs had target genes in cis. As many GWAS loci are associated with complex traits, the vQTLs likely play regulatory roles that mediate the BP associations as well. Interestingly, 8 of the 12 eQTLs were annotated as being both eQTLs and sQTLs, while cis-eQTLs have only a 12% overlap with cis-sQTLs in GTEx^27^. This agreed with the finding that sQTLs in GWAS results display stronger associations with complex traits than variants that exclusively affect gene expression^28,29^.

**Figure 6:**
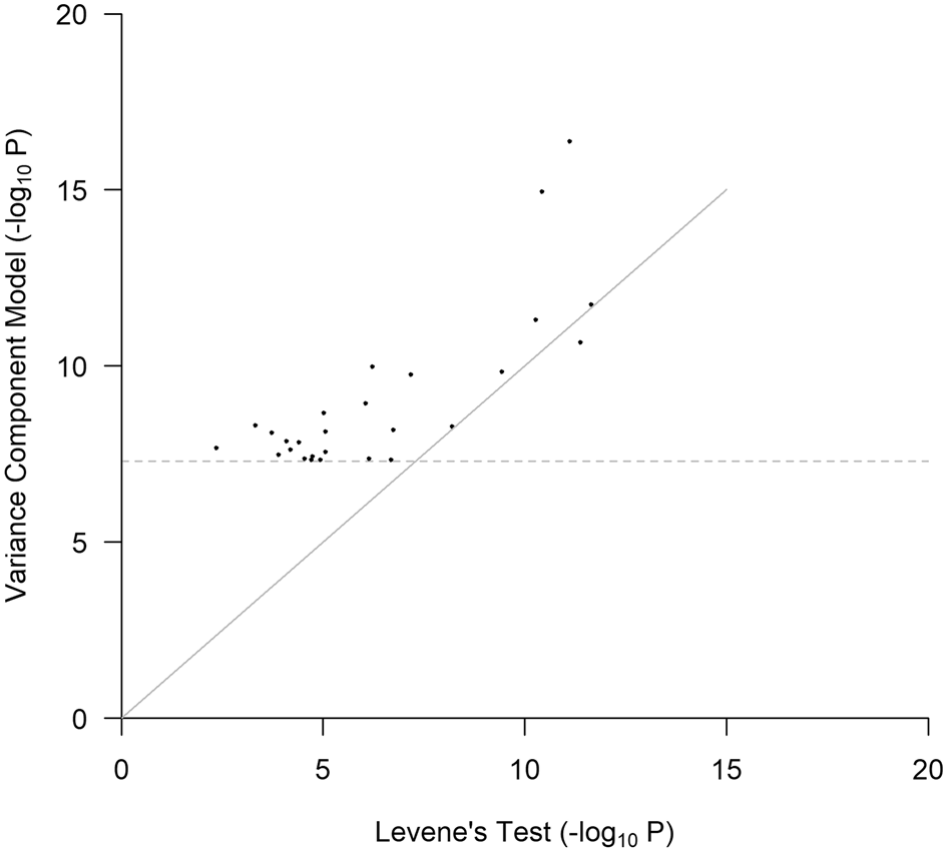
P values of the 27 vQTLs by Levene’s test. The dashed line represents the genome-wide significance level (P = 5 × 10^−8^).

Nonparametric methods are widely available for heteroscedasticity testing and have been used in vQTL analysis. They are not based on particular assumptions about trait distributions and are applicable for analyzing a wide range of quantitative traits. Of the BPs in this work, SBP and DBP approximately follow normal distributions. PP is highly skewed with a long and fat right tail; hence, I used logarithmic transformation. After adjusting for the covariate effects, residuals of SBP, DBP and the transformed PP have skewness values of 0.55, 0.36, 0.03 and kurtosis values of 0.61, 0.25, 0.23, respectively, although their normality tests were rejected given the large sample size of this study. I further conducted Levene’s (Brown-Forsythe) test^30,31^ on the 27 vQTLs identified by the variance component test. SNP genotypes were called those with the largest probabilities and residuals after adjusting for covariate effects, and the SNP main effect was used to test the equality of variance in the three genotype groups. The P values of Levene’s tests and the variance component test are compared in Fig. 6. It can be seen that Levene’s tests provide varying levels of support for the 27 vQTLs. Unsurprisingly, the results of the variance component model were more significant than those of Levene’s test, except for one vQTL. This agrees with the general case that parametric methods are more powerful when their underlying assumptions are approximately satisfied.

## Discussion

I carried out genome-wide vQTL analysis by testing the allelic substitution effects on trait variance and identified 27 vQTLs associated with BPs. Such heteroscedasticity effects explained additional BP variance that was missed by GWASs. The heteroscedasticity effects of the 13 novel vQTLs were larger than their genetic main effects and were not detected by existing GWASs. In addition, 9 novel vQTLs demonstrated almost no genetic main effects, and their P values for testing SNP main effects were larger than 0.05 in this biobank-scale analysis. Complementary to GWAS, vQTL analysis has the potential to discover more variants associated with other complex traits.

On the other hand, if the heteroscedasticity effects harbor some gene–environment or gene–gene interactions, the overall interaction effects appear to be small compared with the additive main effects of GWAS SNPs. The heteroscedasticity effects of the 411 SBP-associated SNPs that were discovered by GWASs added up to 0.52%, compared with the 5.13% attributable to the SNP main effects. For the 20 vQTLs that were associated with SBP in the analysis, the total heteroscedasticity effects and genetic main effects were 0.20% and 0.30%, respectively. This is consistent with that additive main effects are the major sources of genetic variance^32,33^, and detecting gene–environment interactions usually requires much larger sample sizes^34^. Considering the large sample size used in this study, the number and effect sizes of the vQTLs are small. Hence, the gene–environment and gene–gene interactions are unlikely to explain a major part of the “missing heritability”^35^ of BPs.

While I provide statistical evidence supporting the vQTLs of BP traits, the results should be considered preliminary. In particular, I lack independent replication. Because of the so-called “winner’s curse”^36^, the reported effect sizes of the vQTLs in the discovery samples tend to be overestimated. Replication in external datasets would require much larger sample sizes, which implies that meta-analysis is necessary. To the best of my knowledge, methods and software that facilitate meta-analysis of results from variance component analysis are presently lacking. Alternatively, regression analysis of the vQTLs can be performed in the replication samples. Estimated $\tau_{2}$ and $\tau_{3}$, together with their variance and covariance, can be synthesized by the generalized least squares method^37^. In this way, the meta-analysis of vQTL is methodologically equivalent to the meta-analysis that jointly tests SNP main effects and interaction effects^38,39^.

My discovery of the vQTLs is limited by the diversity of population. Samples in the vQTL analysis were restricted to individuals of European ancestry. Since the causal alleles that by chance have reached higher frequencies are more likely to be identified^40^, analyzing samples of a single ancestry not only limits the transferability of results across populations, but results in ascertainment bias and missing the vQTLs that differ among diverse populations. My study is also limited by the geographical and environmental diversity. Complex traits are known to have a strong geographical component involving genetic predisposition and environmental exposure^41^. Effect sizes of the gene-environment interactions may be smaller in the study samples than in geographically and environmentally more diversified samples. Hence, many potential vQTLs could be missed in this study.

In this work, I focused on genome-wide vQTL analysis of BP data in the UK Biobank. The reported vQTLs may include some interaction effects that were not previously identified. As suggested in the literature^2,5,6,8^, vQTL analysis can be used as a screening tool for prioritizing variants that may harbor interaction effects. Factors modulating the genetic effects can be hypothesized and tested thereafter, which is beyond the scope of this paper. Any interactions that can be detected and confirmed will positively contribute to the understanding of complex traits or diseases.

## Data Availability

This research has been conducted using the UK Biobank resource under application number 44080. The genetic and phenotype datasets are not publicly available but can be accessed via the UK Biobank data access process. More details are available at http://www.ukbiobank.ac.uk/register-apply/.
